# Global Fitness Profiling Identifies Arsenic and Cadmium Tolerance Mechanisms in Fission Yeast

**DOI:** 10.1534/g3.116.033829

**Published:** 2016-08-22

**Authors:** Lan Guo, Abantika Ganguly, Lingling Sun, Fang Suo, Li-Lin Du, Paul Russell

**Affiliations:** * Department of Cell and Molecular Biology, The Scripps Research Institute, La Jolla, California 92037; † National Institute of Biological Sciences, Beijing 102206, People’s Republic of China

**Keywords:** arsenic, cadmium, heavy metals, metal toxicity, *Schizosaccharomyces pombe*

## Abstract

Heavy metals and metalloids such as cadmium [Cd(II)] and arsenic [As(III)] are widespread environmental toxicants responsible for multiple adverse health effects in humans. However, the molecular mechanisms underlying metal-induced cytotoxicity and carcinogenesis, as well as the detoxification and tolerance pathways, are incompletely understood. Here, we use global fitness profiling by barcode sequencing to quantitatively survey the *Schizosaccharomyces pombe* haploid deletome for genes that confer tolerance of cadmium or arsenic. We identified 106 genes required for cadmium resistance and 110 genes required for arsenic resistance, with a highly significant overlap of 36 genes. A subset of these 36 genes account for almost all proteins required for incorporating sulfur into the cysteine-rich glutathione and phytochelatin peptides that chelate cadmium and arsenic. A requirement for Mms19 is explained by its role in directing iron–sulfur cluster assembly into sulfite reductase as opposed to promoting DNA repair, as DNA damage response genes were not enriched among those required for cadmium or arsenic tolerance. Ubiquinone, siroheme, and pyridoxal 5′-phosphate biosynthesis were also identified as critical for Cd/As tolerance. Arsenic-specific pathways included prefoldin-mediated assembly of unfolded proteins and protein targeting to the peroxisome, whereas cadmium-specific pathways included plasma membrane and vacuolar transporters, as well as Spt–Ada–Gcn5-acetyltransferase (SAGA) transcriptional coactivator that controls expression of key genes required for cadmium tolerance. Notable differences are apparent with corresponding screens in the budding yeast *Saccharomyces cerevisiae*, underscoring the utility of analyzing toxic metal defense mechanisms in both organisms.

Nonessential metals such as arsenic (As) and cadmium (Cd) are widespread environmental contaminants that pose grave risks to human health (Jarup 2003; Jarup and Akesson 2009). Poisoning by these metals typically arises from inadequate safety measures at industrial sites or exposure of the general population to polluted air, soil, or groundwater. Indeed, contamination of food and water by these metals is a global environmental problem that puts millions of people at risk. Arsenic and cadmium are directly associated with a range of pathologies including cardiovascular dysfunction, renal failure, birth defects, lung disease, and increased risk of cancer. Notably, both metals are listed as Group 1 carcinogens by the International Agency for Research on Cancer (IARC), which means there is strong evidence they cause cancer in humans.

While the health risks associated with arsenic and cadmium exposure are indisputable there remains substantial uncertainty about the molecular mechanisms for their cellular toxicity and carcinogenic activities. Multiple mechanisms have been proposed. (A) Nonessential metals may disrupt homeostasis of chemically similar metals that are biologically essential (Martelli *et al.* 2006). Competitive binding to functional sites in key metalloenzymes may inhibit critical cellular processes (Faller *et al.* 2005; Martelli *et al.* 2006). (B) In the same vein, both arsenic and cadmium are highly reactive with sulfhydryl groups, which may negatively impact the functions of many proteins. (C) Some metal ions are redox-active or have been suggested to activate oxidases to elevate cellular O_2_^•−^ directly (Chou *et al.* 2004). Reactive oxygen species (ROS) acquired by different and nonexclusive mechanisms attack cellular macromolecules such as lipids, protein, and DNA (Avery 2001). (D) Nearly all toxic metals have been proposed to affect cell cycle progression and interfere with specific DNA repair mechanisms (Jin *et al.* 2003; Lee and Singleton 2004; O’Brien *et al.* 2005; Shi *et al.* 2004). (E) Modulation of gene expression by epigenetic modifications and transcription factor deregulation of cell proliferation has also been suggested to play some role in metal toxicity (Beyersmann and Hartwig 2008; Salnikow and Zhitkovich 2008). All these mechanisms may work cooperatively to cause tumorigenesis (Beyersmann and Hartwig 2008) or different mechanisms may be involved based on the toxicological profile of each metal.

There are other important reasons for investigating the cellular effects of arsenic and cadmium. Arsenical drugs are critical for the last line of defense in certain devastating diseases. Notably, the arsenic-containing drug melarsoprol is used to treat African trypanosomiasis, while arsenic trioxide is a chemotherapy drug used to treat acute promyelocytic leukemia (Dilda and Hogg 2007; Fairlamb 2003). Arsenic trioxide is under investigation for treatment of other types of cancer, which further increases the imperative for understanding its curative and toxic effects, as well as mechanisms of tumor resistance (Kritharis *et al.* 2013). On a different research front, there is considerable interest in using plants and microorganisms to clean up soil and water contaminated by arsenic or cadmium (Macek *et al.* 2008). Obviously, a detailed understanding of cellular metal detoxification mechanisms could be crucial for engineering organisms to enhance bioremediation strategies.

Eukaryotes have evolved multiple defense strategies to cope with exposure to toxic metals. Detoxification and tolerance mechanisms typically consist of reduction of metal uptake, enhanced extrusion, sequestration within vacuoles, and chelation by metal-binding proteins and polypeptides (Mendoza-Cozatl *et al.* 2005; Wysocki and Tamas 2010). Signal transduction pathways coordinate these responses leading to rapid reprograming of cellular transcriptome, proteome, and metabolome profiles. Cellular responses to environmental stresses have been most intensively investigated in the budding yeast *Saccharomyces cerevisiae*, culminating in global deletome or functional profiling screens to assess the importance of the ∼4700 nonessential genes in determining metal resistance. These screens identified nearly 600 genes required for arsenic resistance and >1000 required for cadmium resistance, implicating many cellular processes in metal detoxification and tolerance (Haugen *et al.* 2004; Jin *et al.* 2008; Ruotolo *et al.* 2008; Serero *et al.* 2008; Thorsen *et al.* 2009; Zhou *et al.* 2009). However, the concordance between screens was unexpectedly low, on the order of ∼10–20% when individual screens were compared. This relatively poor overlap could reflect a lack of saturation in the screens as well as a number of experimental variations, such as screening on liquid *vs.* solid media or using different assays to measure toxicity.

Owing to its comparable experimental advantages and its distant evolutionary relationship to *S. cerevisiae*, the fission yeast *Schizosaccharomyces pombe* has proven to be an outstanding model organism for investigating basic cellular processes. With the smallest genome among the commonly used eukaryotic model systems (Wood *et al.* 2002), coupled with cellular properties more commonly associated with complex multicellular organisms, fission yeast is a useful counterpoint for investigating biological mechanisms that are broadly conserved among eukaryotes. Approximately 10% of fission yeast genes lack budding yeast homologs and yet are conserved in other eukaryotic species, including humans. In some cases the fission yeast stress response mechanisms more closely resemble those in plants or mammals. Perhaps most notable in the context of metal toxicity, fission yeast and plants share the ability to synthesize a glutathione oligomer known as phytochelatin, which is an especially effective chelator of cadmium (Clemens *et al.* 1999; Gacto *et al.* 2003). We previously reported using an early version of the fission yeast deletome to screen mutants arrayed on agar plates for cadmium sensitivity (Kennedy *et al.* 2008). Here, we use deep sequencing of barcodes in deletion cassettes with an expanded version of the deletome to screen both cadmium and arsenic sensitivity, yielding sensitive quantitative measurements for >2900 mutants representing about 85% of the haploid deletome. The data reveal both shared and metal-specific defense mechanisms for arsenic and cadmium, and reveal the relative importance of these mechanisms for each toxic metal. There are both striking similarities and differences with comparable screens of the budding yeast *S. cerevisiae*, indicating evolutionary divergence of some defense mechanisms and potential species-specific genetic redundancies within conserved pathways.

## Materials and Methods

### Strains, media, and reagents

The *S. pombe* haploid deletion library from Bioneer has genes deleted with the KanMX4 cassette in the genetic background *h*^+^ *leu1-32 ura4-D18 ade6-M210/M216*. Our screens used Version 1.0 or an updated library consisting of 3004 mutants (Kim *et al.* 2010). Unless noted, all mutants used in subsequent analyses were from the Bioneer deletion collection. Cells were grown in YES media (yeast extract with glucose and supplements) under standard growth conditions (Forsburg and Rhind 2006; Moreno *et al.* 1991), with or without cadmium sulfate hydrate (Sigma 255513, St. Louis) or sodium arsenite (Sigma S-7400). Edinburgh minimal media-2 (EMM2) was used to assess amino acid auxotrophy (Forsburg and Rhind 2006).

### Microculture growth assay

To find physiologically relevant cadmium and arsenic concentrations that were modestly toxic we monitored the growth of wild-type cells in YES media containing 0–800 μM NaAsO_2_ or 0–500 μM CdSO_4_. Briefly, 100-μl cultures of midlog cells at ∼0.2 OD_600_ were aliquoted into a flat bottom 96-well plate and incubated at 30° with occasional shaking. An automated microplate reader (VERSAmax, Molecular Devices, Sunnyvale, CA) monitored OD_600_ every 30 min for 16 hr. The values generated were the average of three technical repeats (Supplemental Material, Figure S1A). Toxicant concentrations that caused a 10–20% decrease in growth rates, 5 μM CdSO_4_ and 100 μM NaAsO_2_, were used as the initial conditions for the functional profiling screens.

### Screening and confirmation of metal-sensitive mutants

Detailed procedures for deletion library pool construction, deep sequencing, and barcode data analysis were as described (Han *et al.* 2010). Briefly, frozen aliquots of the pooled deletion strains were recovered in YES media and allowed to grow for 5 hr at 30°. Samples were harvested and designated as the 0 time point sample. For metal toxicity experiments, the metal compounds were added at the 0 time point and cells allowed to grow for ∼5 generations. Cells were lysed in TE buffer (10 mM Tris-HCl, 1 mM EDTA, pH 8.0), genomic DNA was extracted, and the barcodes were amplified with Ex Taq DNA polymerase (TaKaRa, Otsu, Shiga, Japan). PCR products were purified and mixed in equal molar amounts to use as the Illumina sequencing template. Standard single-end sequencing primers were used for 42 cycles of sequencing in an Illumina Genome Analyzer II.

Barcode sequencing data have been deposited at the National Center for Biotechnology Information (NCBI) Sequence Read Archive (http://www.ncbi.nlm.nih.gov/sra/) under the accession number SRX2022570. The data are composed of ten runs belonging to three batches of experiments. Batch one includes untreated control (run SRR4031062, uptag index is CGAT and dntag index is TATA), 5 µM CdSO_4_ treatment (run SRR4031053, uptag index is AGCT and dntag index is TCGA), and 100 µM NaAsO_2_ treatment (run SRR4031054, uptag index is CTA and dntag index is GAT); batch two includes untreated control (run SRR4031060, uptag index is CGAT and dntag index is TATA), 5 µM CdSO_4_ treatment (run SRR4031055, uptag index is CAGT and dntag index is ATTA), 100 µM NaAsO_2_ treatment (run SRR4031056, uptag index is TAAT and dntag index is AGGA), and 200 µM NaAsO_2_ treatment (run SRR4031057, uptag index is ATC and dntag index is GCG); batch three includes untreated control (run SRR4031061, uptag index is ACGT and dntag index is GACT), 3 µM CdSO_4_ treatment (run SRR4031058, uptag index is TGCA and dntag index is GTCA), and 5 µM CdSO_4_ treatment (run SRR4031059, uptag index is TGGT and dntag index is GTGT).

### Calculation of growth inhibition score

A growth inhibition (GI) score was calculated for each mutant in the library to determine its toxin sensitivity (Han *et al.* 2010). Briefly, a normalized log2 ratio of barcode sequencing counts in control *vs.* treatment samples was calculated for each mutant. This calculation normalizes values for slow-growing mutants. The ratio was divided by the number of cell doublings (five for the screens analyzed here) to obtain the GI score. For treatment-sensitive mutants, we expect GI scores higher than 0 because of the depletion of these mutants from the deletion pool under the treatment condition. The mutants that completely failed to grow under the treatment condition are expected to have GI scores around 1. To select treatment-sensitive mutants, a robust *Z*-score was calculated for each gene, which is the deviation of its GI score from the median GI score expressed in the number of the normalized interquartile range (NIQR). Tail area-based false discovery rate (FDR) values were calculated from the robust Z-scores using the software fdr tool Version 1.2.8 (Strimmer 2008). We performed four independent screens with cadmium and three with arsenic. One screen for each compound was performed with Version 1.0 of the Bioneer haploid collection, with the subsequent screens performed with an updated library consisting of 3004 mutants. Consequently a few hundred genes only have a GI score for three of four screens for cadmium sulfate and two of three screens for sodium arsenite. Overall we were able to assign a GI score to a total of 2902 genes from the Bioneer deletion library.

We initially identified mutations that satisfied the FDR cutoff of 0.1 in all screens for each treatment. For these mutants expressivity (high, medium, or low) was determined from GI scores using arbitrary cutoff values. Subsets of mutants failed to meet this stringent FDR cutoff and yet appeared to be sensitive in at least one screen. These mutants were picked out from the library and further tested by spot assay analysis. Mutants from YES agar plates supplemented with 150 mg/l G418 were incubated in YES liquid media in a 96-well plate and grown at 30° for 2–3 d to reach saturation density. Cultures were then diluted 20-fold (OD_600_ ∼ 0.4) and incubated for another 3 hr. Ten-fold serial dilutions of the cell cultures were spotted onto YES agar with or without NaAsO_2_[As(III)] or CdSO_4_[Cd(II)]. Cadmium sensitivity was assessed on plates containing 5, 25, 100, or 200 μM CdSO_4_, while arsenic sensitivity was measured on plates containing 25, 50, 100, or 200 μM NaAsO_2_. Metal sensitivity was assessed by visual inspection. Note that cadmium and arsenic appear to be more toxic at lower concentrations in liquid media compared to agar media, thus the heavy metal concentrations used for the spot dilution assays were empirically determined. For these mutants expressivity (high, medium, or low) was based on the lowest concentration of metal to which the mutant was clearly sensitive. Note that these expressivity categories are not directly comparable to those determined using GI scores.

### RNA purification and mRNA expression levels

For mRNA expression analysis, log-phase cultures were grown to 0.6–0.8 OD_600_ at 30° and treated with 5 μM CdSO_4_ or 200 μM NaAsO_2_ for 2 hr. Aliquots were harvested by centrifugation, washed with ice-cold water, and frozen at −80°. Total RNA was extracted using QIAGEN RNeasy Plus Mini Kit (74134) following the manufacturer’s protocol. Quantitative reverse transcription PCR (RT-qPCR) was performed with 60 ng of total RNA using iScript One-Step RT-PCR Kit with SYBR Green (Bio-Rad, 170-8892) on a CFX Connect Real-Time Detection System (Bio-Rad). All data were normalized with *act1* mRNA as the internal control and mRNA from wild type without toxin treatment as the calibrator. Primers used for all other genes are listed in Table S1.

### GO term enrichment and YOGY

Gene ontology (GO) term enrichment analysis was performed using the web-based tool AnGeLi (Bitton *et al.* 2015). Cluster 3.0 was used for hierarchical clustering analysis and Java TreeView was used for visualization of the cluster analysis. Pair-wise average-linkage and uncentered correlation was chosen for analysis. Online software YOGY (http://www.bahlerlab.info/YOGY/) (Penkett *et al.* 2006) was used for retrieving homologous proteins from humans and *S. cerevisiae.*

### Data availability

The authors state that all data necessary for confirming the conclusions presented in the article are represented fully within the article.

## Results and Discussion

### Global fitness profiling for cadmium and arsenic sensitivity

Parallel mutant phenotyping by barcode sequencing (Bar-seq) (Han *et al.* 2010) was used to assay pooled haploid deletion mutants of fission yeast exposed to cadmium sulfate or arsenite. To optimize assessment of both healthy mutants and those having a partial growth defect we used metal or metalloid concentrations that caused a 10–20% growth inhibition in wild-type cells (Figure S1A). Using microculture growth assays we selected concentrations of 3–5 μM CdSO_4_ [Cd(II)] and 100–200 μM NaAsO_2_ [As(III)] for the parallel mutant phenotyping. Pooled libraries consisting of ∼3000 mutants representing ∼80% of the haploid deletome were grown for ∼5 generations in the presence or absence of the compounds. We performed four screens with cadmium and three with arsenite. Growth inhibition scores were obtained for 2902 mutants (see *Materials and Methods*) (Table S2). Correlation coefficients for independent biological replicates ranged from 0.52 to 0.8 (Figure S1, B and C). Mutants that were significantly sensitive in all screens for either compound were identified (FDR cutoff of 0.1), yielding 51 mutants for CdSO_4_ and 83 for NaAsO_2_. Additionally, mutants that had significant GI scores in a subset of screens were rescreened by spot dilution assays on agar plates. Mutants that displayed increasing toxin sensitivity on plates containing ≤100 μM CdSO_4_ or ≤200 μM NaAsO_2_ were considered toxin sensitive. Ultimately we identified 106 cadmium-sensitive and 110 arsenite-sensitive mutants (Table 1). The mutants were ranked according to their average GI scores and further divided into high, medium, or low expressivity groups when tested by parallel mutant phenotyping in liquid media or by spot dilution assays on agar plates (Table S3 and Table S4). Most of the identified genes have homologs in *S. cerevisiae* (∼86%) and humans (∼72%) (Table 1). However, the majority of the corresponding *S. cerevisiae* homologs were not identified in deletome screens for cadmium or arsenic sensitivity (Table 1, Table S3, and Table S4), indicating significantly different genetic requirements for heavy metal detoxification in the two organisms.

**Table 1:** Summaries of cadmium and arsenic functional profiling screens

Metal	Method	Concentration (μM)	Verified	Total	*S. cerevisiae* Orthologs	Human Orthologs	*S. cerevisiae* Ortholog Sensitivity
Cd(II)	Bar-seq	3, 5	51	106	90 (85%)	75 (70%)	34/90 (38%)
	Spot Assay	5–100	55				
As(V)	Bar-seq	100, 200	83	110	96 (87%)	70 (63%)	20/96 (21%)
	Spot Assay	25–200	27				

*S. cerevisiae* and *H. sapiens* orthologs were derived from the online tool YOGY (Penkett *et al.* 2006). *S. cerevisiae* orthologs implicated in heavy metal resistance were found among 726 genes identified in four cadmium deletome screens (Jin *et al.* 2008; Ruotolo *et al.* 2008; Serero *et al.* 2008; Thorsen *et al.* 2009) or 486 genes in three arsenic deletome screens (Haugen *et al.* 2004; Jin *et al.* 2008; Thorsen *et al.* 2009).

### Shared critical pathways required for arsenite and cadmium tolerance

**Figure 1:**
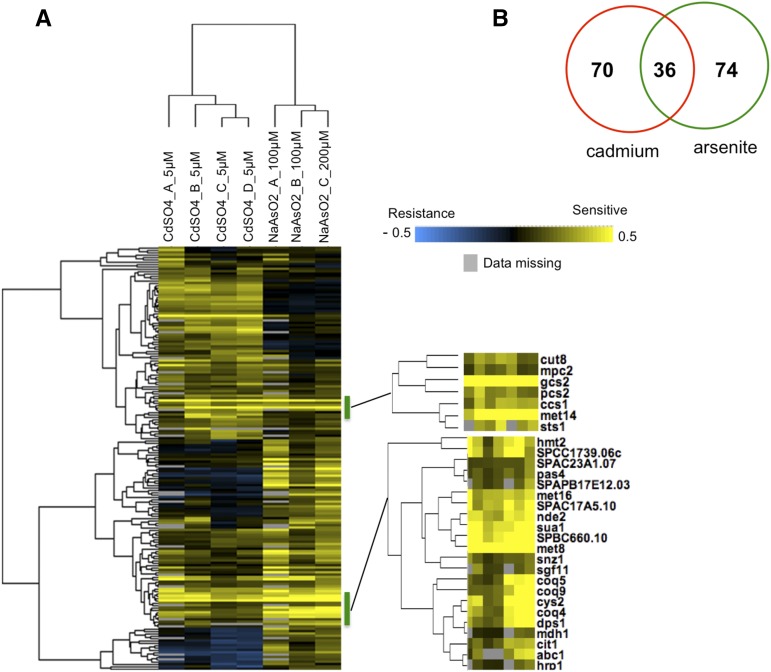
Hierarchical clustering analysis of mutants identifies genes required for both cadmium and arsenite resistance. (A) Clustergram involving all mutants sensitive to arsenite or cadmium. Zoom view (right panel) indicates specific clusters that are shared by both arsenite and cadmium resistance. We identified a critical requirement for genes involved in the cysteine biosynthesis pathway, and its utilization in synthesizing glutathione and phytochelatin in providing tolerance to arsenite and cadmium exposure. “Data missing” indicates situations in which a GI score could not be calculated for the indicated gene. (B) Venn diagram showing a high degree of overlap (p value = 1.35e−35) between genes identified for arsenite (green) or cadmium (red) tolerance as determined by two-tailed Fisher’s exact test).

The screens identified 36 genes that are required for both cadmium and arsenite resistance (Figure 1, A and B and Table S5). This overlap is highly significant with a p value = 1.35e−35 when compared among the 5135 protein-encoding genes of fission yeast. Note that unless otherwise stated statistical analyses were two-sided Fisher’s exact tests with p value corrected according to FDR and evaluated given an α of 0.01 (Benjamini and Hochberg 1995). Furthermore, as judged by GI scores, five of the 10 most sensitive mutants were shared in the cadmium and arsenite lists (*met14*, *met8*, *gcs2*, *sua1*, and *mef2*). Thus there is very significant overlap in the genes that are most critical for survival of cadmium and arsenite exposure, suggesting that shared mechanisms detoxify these chemically dissimilar heavy metals.

**Figure 2:**
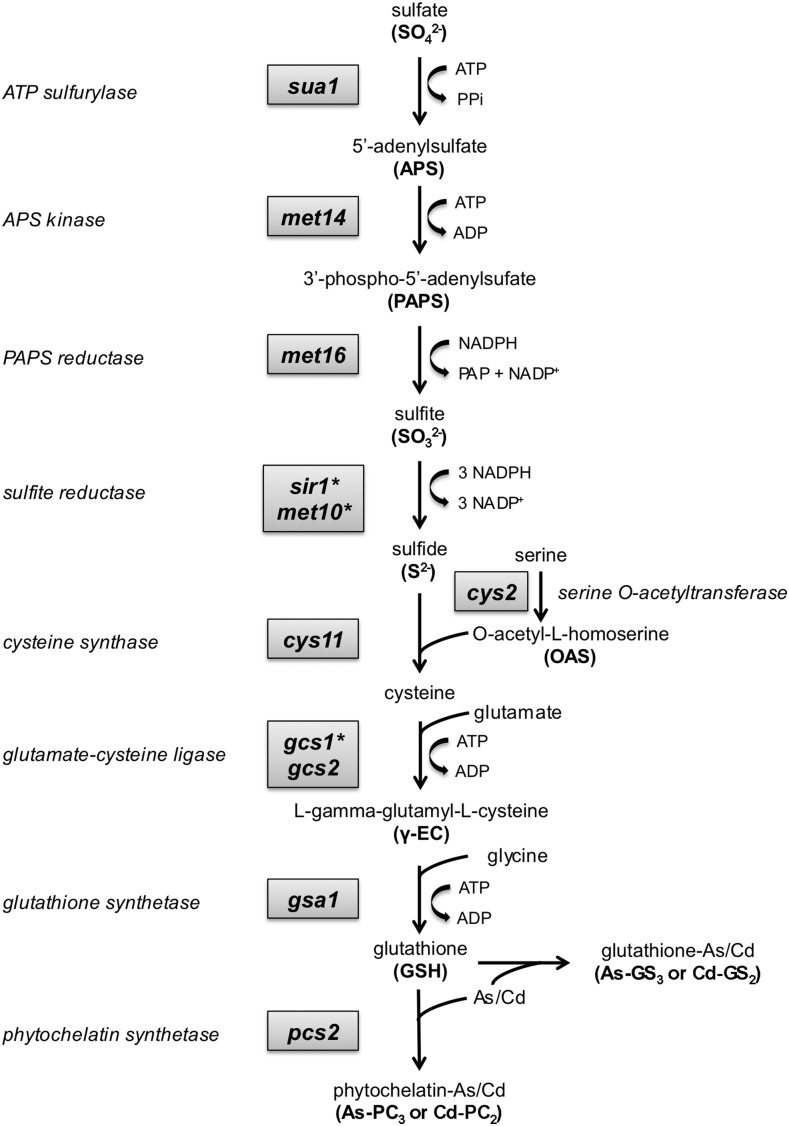
Sulfate assimilation leading to glutathione and phytochelatin synthesis is critical for cadmium and arsenite resistance in *S. pombe*. The diagram illustrates the biochemical pathway starting with sulfate and leading to cysteine biosynthesis, formation of glutathione and phytochelatin, and chelation of arsenic and cadmium by these cysteine-rich peptides. All genes of this pathway were identified in the arsenic and cadmium functional profiling screens, with the exception of three genes indicated by an asterisk (*sir1*, *met10*, and *gcs1*) that were absent from the deletome library used in these studies. These three genes were previously shown to be critical for cadmium resistance (Kennedy *et al.* 2008).

We used the web-based tool AnGeLi to identify GO terms and other properties that were enriched among the 36 genes that are required for both cadmium and arsenite resistance (Bitton *et al.* 2015). This analysis highlighted three parent GO biological process categories: (1) sulfur compound metabolic process, particularly biosynthesis of sulfur amino acids (cysteine and methionine) and glutathione; (2) cofactor biosynthesis, notably ubiquinone and siroheme biosynthesis; and (3) cellular response to metal ions, especially phytochelatin biosynthesis (Table 2). These GO terms accounted for 19 of the 36 genes required for tolerance of both cadmium and arsenite. As discussed in detail below, 18 of these genes are involved in a series of biochemical steps starting with sulfate assimilation and leading to the biosynthesis of glutathione and phytochelatin (Figure 2), which chelate cadmium and arsenic (Mendoza-Cozatl *et al.* 2005).

**Table 2:** Summary of GO categories enriched by mutants sensitive to arsenite and cadmium (background is protein-encoding genes)

GO Category	Fold Enrichment	p Value	List Frequency (36 genes)	Background Frequency (5135 genes)	Genes
**Biological process**					
Sulfur compound metabolic process	15	3.40e−07	27.78% (10)	1.81% (93)	*SPCC1739.06c*, *cys2*, *gcs2*, *gsa1*, *met14*, *cys11*, *hmt2*, *sua1*, *met16*, *met8*
*Sulfur amino acid metabolic process*	25	3.11e−05	16.67% (6)	0.66% (34)	*SPCC1739.06c*, *cys2*, *met14*, *cys11*, *met16*, *sua1*
*Glutathione biosynthesis*	104	2.18e−04	8.33% (3/36)	0.08% (4)	*gcs2*, *gsa1*, *hmt2*
Cofactor biosynthetic process	12	5.30e−5	22.22% (8)	1.85% (95)	*snz1*, *SPCC1739.06c*, *coq3*, *nde2*, *coq5*, *dps1*, *coq4*, *coq9*, *met8*
*Ubiquinone biosynthetic process*	56	6.30e−06	13.89% (5)	0.25% (13)	*coq9*, *coq4*, *coq3*, *coq5*, *dps1*
*Siroheme biosynthetic process*	139	4.19e−03	5.56% (2)	0.04% (2)	*SPCC1739.06c*, *met8*
Response to metal ion	36	5.02e−04	11.11% (4)	0.31% (16)	*gsa1*, *hmt2*, *pcs2*, *ccs1*
*Phytochelatin biosynthetic process*	104	2.18e−04	8.33% (3)	0.08% (4)	*gsa1*, *hmt2*, *pcs2*
**Cellular component**					
Mitochondrion	3	5.73e−03	41.67% (15)	14.10% (724)	*SPBC660.10*, *ccs1*, *cem1*, *coq3*, *coq4*, *coq5*, *coq9*, *cys11*, *cys2*, *dps1*, *hmt2*, *mpc2*, *nde2*, *sty1*, *sua1*
*Mitochondrial inner membrane*	7	2.02e−03	22.22% (8)	3.17% (163)	*ccs1*, *coq3*, *coq5*, *coq9*, *dps1*, *mpc2*, *nde2*, *sty1*

AnGeLi highlighted a number of annotated mutant phenotypes in PomBase that were highly enriched among the 36 genes required for arsenite and cadmium tolerance (Table 3). Notably 18 mutants were “sensitive to cadmium” (p = 9.50e−12), 16 were “sensitive to hydrogen peroxide” (p = 5.36e−13), 13 displayed “abolished cell population growth on glycerol carbon source” (p = 8.19e−13), and four were “growth auxotrophic for cysteine” (p = 3.89e−07).

**Table 3:** Summary of common phenotypes associated with mutants sensitive to arsenite and cadmium (background is protein-encoding genes)

Phenotypes	Fold Enrichment	p Value	List Frequency (36 genes)	Background Frequency (5135 genes)	Genes
Sensitive to hydrogen peroxide	16	5.36e−13	44.44% (16)	2.75% (141)	*mms19*, *met16*, *coq4*, *met14*, *sty1*, *cuf1*, *met8*, *sua1*, *hmt2*, *cys11*, *SPBC660.10*, *dps1*, *coq3*, *SPCC1739.06c*, *coq5*, *aah3*
Abolished cell population growth on glycerol carbon source	27	8.19e−13	36.11% (13)	1.32% (68)	*mms19*, *met16*, *coq4*, *met14*, *sty1*, *cuf1*, *met8*, *sua1*, *cys11*, *SPBC660.10*, *dps1*, *coq3*, *coq5*
Sensitive to cadmium	10	9.50e−12	50.00% (18)	4.85% (249)	*mms19*, *met16*, *coq4*, *met14*, *cut8*, *sty1*, *cuf1*, *gsa1*, *pcs2*, *met8*, *SPAC9.02c*, *sua1*, *hmt2*, *cys11*, *coq3*, *SPCC1739.06c*, *coq5*, *aah3*
Growth auxotrophic for sulfur-containing amino acid	57	3.89e−07	16.67% (6)	0.29% (15)	*mms19*, *met16*, *met14*, *cys2*, *sua1*, *cys11*

### Sulfur assimilation leading to cysteine, glutathione, and phytochelatin biosynthesis is critical for arsenic and cadmium resistance

As mentioned above, the functional profiling screens identified sulfate assimilation leading to cysteine, glutathione, and phytochelatin biosynthesis as being crucial for arsenite and cadmium resistance. Indeed, most heavy metals are characterized by their high affinity for protein sulfhydryl groups, which typically results in the inhibition of protein functions (Mendoza-Cozatl *et al.* 2005; Thorsen *et al.* 2009). A subset of genes encoding key enzymes associated with sulfate assimilation and cysteine biosynthesis is up-regulated during cadmium exposure (Chen *et al.* 2003; Harrison *et al.* 2005). In fact, mutants corresponding to almost all of the genes encoding enzymes required to transform sulfate into phytochelatin were identified in the screens (Figure 2). The exceptions were three mutants that were absent from this screen (*sir1*Δ, *met10*Δ, and *gcs1*Δ), although we had previously shown that these mutants are extremely sensitive to cadmium (Kennedy *et al.* 2008). These genes are likely to also be essential for arsenic resistance. This remarkable coverage underscores the quality of the functional profiling screens and the extreme importance of synthesizing glutathione and phytochelatin to detoxify cadmium and arsenic.

### Methionine biosynthesis is not required for As/Cd resistance

**Figure 3:**
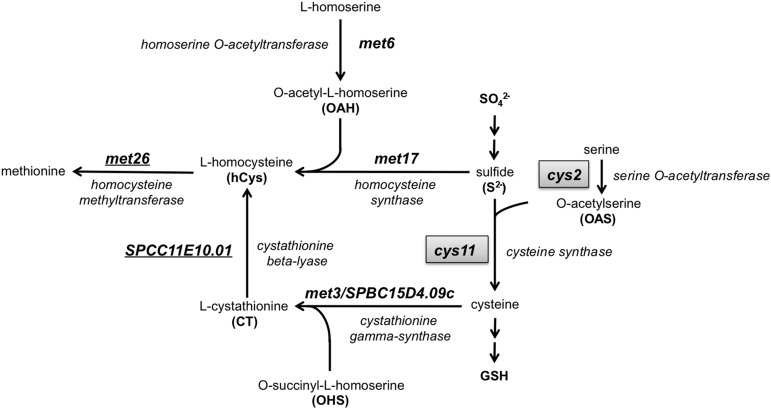
Methionine biosynthesis is not required for cadmium and arsenite resistance in *S. pombe*. The diagram illustrates the methionine biosynthesis pathway in fission yeast. Deletome mutants of genes specifically involved in methionine but not cysteine biosynthesis such as *met6* and *met17* were found to be insensitive to cadmium and arsenic in the functional profiling screens. In contrast genes such as *cys2* and *cys11* (highlighted in boxes), required for cysteine biosynthesis, were found to be essential for cadmium and arsenic resistance.

The gene encoding cysteine synthase Cys11, which is specifically required for cysteine but not methionine biosynthesis, was identified among the 36 required for arsenite and cadmium resistance (Figure 3). In contrast, functional profiling did not detect any As/Cd sensitivity for mutants that lacked genes that are predicted to be specifically involved in methionine biosynthesis, such as homocysteine synthase Met17, homoserine *O*-acetyltransferase Met6, and cystathionine γ-synthase Met3/SPBC15D4.09c (Figure 3). These data, along with evidence that fission yeast has a marginal ability only to synthesize cysteine from methionine (Brzywczy *et al.* 2002), are consistent with cysteine but not methionine being an essential precursor for synthesis of glutathione and phytochelatin.

### Cys2 is a serine *O*-acetyltransferase required for cysteine biosynthesis

Functional profiling revealed that Cys2 is essential for As/Cd tolerance (Table S5). Cys2 has greatest sequence homology to homoserine *O*-acetyltransferases, predicting that it should be involved with methionine biosynthesis. However, as noted previously, Met6 is also predicted to be a homoserine *O*-acetyltransferase (Ma *et al.* 2007). *S. cerevisiae* homoserine *O*-acetyltransferase Met2 is significantly more similar to Met6 than Cys2 in *S. pombe*, suggesting that in *S. pombe* Met6 is more likely the authentic homoserine *O*-acetyltransferase (Figure 3). Furthermore, *met6*Δ mutants require methionine supplementation for growth on defined minimal medium whereas *cys2*Δ cells require cysteine supplementation (Ma *et al.* 2007). Thus our data showing that *cys2*Δ but not *met6*Δ cells are highly sensitive to As/Cd toxicity, as well as our data indicating that methionine biosynthesis is not required for cadmium or arsenic resistance, support the notion that Cys2 is actually a serine *O*-acetyltransferase that is specifically essential for cysteine biosynthesis (Figure 3).

### Mms19-dependent cytosolic iron-sulfur protein assembly into Sir1-Met10 sulfite reductase is essential for As/Cd resistance

We previously found that Mms19/SPAC1071.02 is essential for cadmium tolerance in fission yeast (Kennedy *et al.* 2008). Our current screens confirm this result and additionally show a critical role for Mms19 in arsenite tolerance (Table S5). In *S. cerevisiae* the Mms19 ortholog, known as Met18, is a late-acting component of the cytosolic iron-sulfur protein assembly (CIA) machinery. Met18^Mms19^ associates with Cia1 and Cia2 to form the CIA machinery that directs Fe-S cluster incorporation into a subset of Fe-S-dependent proteins involved in DNA replication and repair, transcription, and telomere maintenance (Gari *et al.* 2012; Stehling *et al.* 2012). The fission yeast homologs of Cia1 and Cia2, SPAC806.02c and SPAC144.16, are essential for viability (Kim *et al.* 2010), hence they were not included in our deletome study. However, fission yeast Mms19 is also found as a subunit of a Rik1-Raf2/Dos2-Mms19-Cdc20 protein complex that is involved in DNA replication, siRNA production, and heterochromatin assembly (Li *et al.* 2011). In the Rik1-Raf2/Dos2-Mms19-Cdc20 protein complex, the gene encoding the heterochromatin silencing protein Rik1 was represented in the deletome library but this *rik1*Δ mutant was insensitive to both cadmium and arsenite (Table S2).

**Figure 4:**
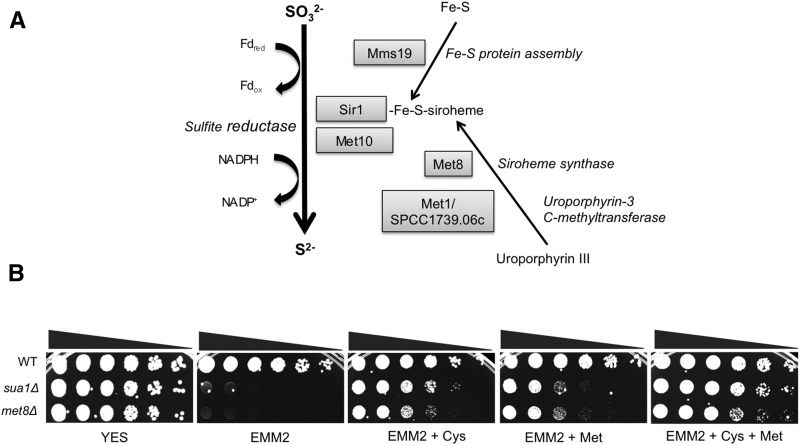
Mms19-dependent cytosolic iron–sulfur protein assembly and siroheme synthesis are required for cadmium and arsenite resistance. (A) The diagram illustrates the reduction of sulfite to sulfide that is catalyzed by the enzyme sulfite reductase. An Fe–S cluster and siroheme covalently couple with the enzyme to help catalyze the reaction. The requirement for Mms19 in As/Cd resistance most likely reflects its role in promoting Fe–S cluster assembly into the Sir1-Met10 sulfite reductase protein complex. The requirements for siroheme synthase Met8 and the predicted uroporphyrin-3 *C*-methyltransferase Met1/SPCC1739.06c for cadmium and arsenite resistance most likely reflect their roles in providing siroheme for Sir1-Met10 sulfite reductase. (B) The inability of *met8*Δ and *sua1*Δ mutants to grow on synthetic defined media (EMM2) is rescued by supplementation with cysteine.

Interestingly, Fe-S cluster incorporation is also required for the sulfite reductase activity of the Met5–Met10 protein complex in budding yeast, which supplies sulfur for methionine biosynthesis in a CIA-dependent manner (Stehling *et al.* 2012) and studies in fission yeast also confirm the methionine auxotrophy of *mms19*Δ mutants (Li *et al.* 2011). Although the sulfite reductase Sir1 was not detected in our current deletome screen, our previous study did find it to be essential for cadmium resistance in fission yeast (Kennedy *et al.* 2008; Perego *et al.* 1997). Moreover, our previous functional profiling study found that deletion of Met10/SPCC584.01c, which encodes the predicted sulfite reductase NADPH flavoprotein subunit homologous to ScMet10, also causes acute cadmium sensitivity (Kennedy *et al.* 2008). These findings along with the obvious importance of sulfate assimilation pathway in As/Cd resistance supports the idea that the requirement for Mms19 in As/Cd resistance most likely reflects its role in promoting Fe-S cluster assembly into the Sir1-Met10 sulfite reductase protein complex as opposed to its role in heterochromatin maintenance (Figure 4A).

### Siroheme requirement for As/Cd resistance

Sulfite reductase is an interesting enzyme in that it uses siroheme, an iron-containing isobacteriochlorin, alongside a [4Fe-4S] cluster to perform the six-electron reduction of sulfite to sulfide (Crane *et al.* 1995) (Figure 4A). This requirement for siroheme likely explains why our previous screen uncovered a critical requirement for the predicted siroheme synthase Met8 in cells exposed to cadmium (Kennedy *et al.* 2008). The present screen confirmed this result and also revealed that Met8 is essential for arsenite resistance. To formally test this idea we determined whether *met8*Δ mutants require cysteine or methionine for growth in EMM2 media. As predicted, we found that *met8*Δ cells displayed a severe growth defect in EMM2 media that was rescued by addition of cysteine and methionine (Figure 4B). For this experiment we included the control of *sua1*Δ cells that lack sulfate adenylate transferase activity that is critical for biosynthesis of sulfur-containing amino acids (Figure 2). As predicted from previous studies that fission yeast has the enzymes required for converting cysteine into methionine via cystathionine and homocysteine (Brzywczy and Paszewski 1994), we observed that addition of cysteine alone was sufficient to permit growth of *met8*Δ mutants on EMM2 media (Figure 4B). However, we also found that addition of methionine was sufficient to support growth of *met8*Δ mutants on EMM2 media. This finding was unexpected because fission yeast was reported to lack the cystathionine β-synthase and cystathionine γ-lyase enzymes that convert methionine into cysteine (Brzywczy and Paszewski 1994). However, we note that a previous study found that addition of cysteine or methionine to EMM2 was sufficient to rescue growth of *met16*Δ cells, which lack a phosphoadenosine phosphosulfate reductase required for sulfate assimilation (Garcia-Santamarina *et al.* 2013). Furthermore we found that addition of methionine partially suppressed the growth defect of *sua1*Δ cells (Figure 4B). Thus it appears that at least in certain circumstances fission yeast displays limited ability to use methionine to make cysteine.

We had previously identified a predicted uroporphyrin-3 *C*-methyltransferase encoded by *met1/SPCC1739.06c* as being critical for cadmium tolerance (Kennedy *et al.* 2008). This enzyme is essential for synthesis of precorrin-2, which is converted into siroheme by Met8 siroheme synthase (Raux *et al.* 1999; Stroupe *et al.* 2003). Our present screen confirmed the requirement for *met1* in cadmium tolerance and further found that it is also crucial for arsenite resistance. These finding are consistent with the siroheme requirement for sulfite reductase activity and its critical role in synthesis of cysteine leading to glutathione and phytochelatin synthesis (Figure 4A).

### Sulfide-quinone oxidoreductase Hmt2 is critical for both cadmium and arsenite resistance

**Figure 5:**
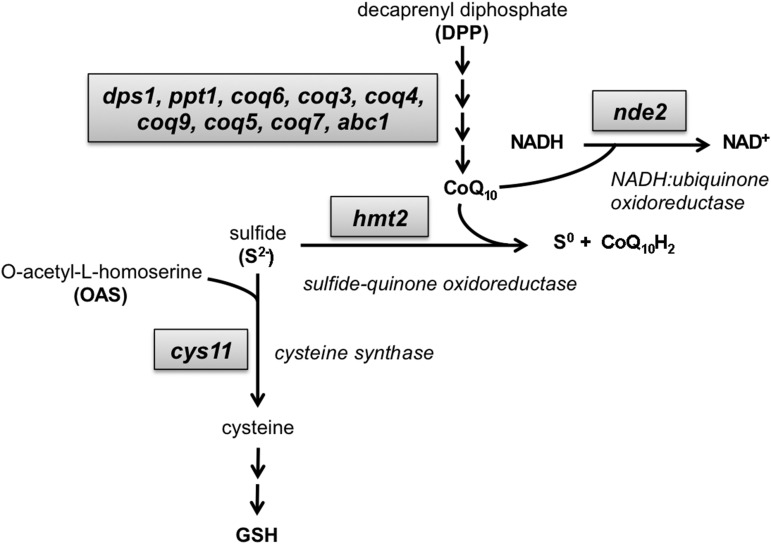
Sulfide-quinone oxidoreductase Hmt2, CoQ_10_ biosynthesis, and NADH:ubiquinone oxidoreductase are critical for both cadmium and arsenite resistance. The schematic illustrates the genes involved in CoQ_10_ biosynthesis as well as the utilization of quinone as an electron acceptor when converting sulfide into sulfur that involves the oxidoreductase Hmt2. Our screens highlighted the mitochondrial sulfide-quinone oxidoreductase Hmt2 as being critical for cadmium and arsenic tolerance, which most likely also explains why our screens identified multiple subunits of the ubiquinone/coenzyme Q_10_ (CoQ_10_) biosynthesis pathway as being important for cadmium or arsenic tolerance.

In agreement with previous studies (Kennedy *et al.* 2008; Vande Weghe and Ow 1999), our current screen highlighted the mitochondrial sulfide-quinone oxidoreductase Hmt2 as being critical for cadmium tolerance (Table S5). Our studies further revealed that Hmt2 is also crucial for arsenite resistance. Sulfide-quinone oxidoreductase converts sulfide (S^2−^) into elemental sulfur (S^0^) and in doing so ensures that sulfide does not accumulate to abnormal levels (Figure 5). We previously reported that *hmt2*Δ cells grown in the presence of cadmium accumulate high amounts of hydrogen sulfide and abnormal CdS deposits (Kennedy *et al.* 2008). High concentrations of sulfide cause cellular toxicity by inhibiting mitochondrial functions. However, the extreme cadmium sensitivity of *hmt2*Δ cells is more likely explained by a defect in production of phytochelatin–cadmium complexes (Vande Weghe and Ow 2001). The inability to accumulate PC–Cd complexes in *hmt2*Δ cells was traced to defects in up-regulating glutathione synthesis leading to an inability to increase phytochelatin synthesis. Abnormally high levels of sulfide resulting in depletion of free Cd^2+^ has been proposed to prevent up-regulation of glutathione production although the mechanism remains unclear (Vande Weghe and Ow 2001). There is uncertainty as to whether *hmt2*Δ cells are sensitive to other metals such as copper and zinc (Vande Weghe and Ow 2001), but our studies clearly show they are quite sensitive to arsenite. As our studies also show that phytochelatin synthesis is critical for arsenite tolerance, it seems reasonable to presume that the underlying causes of arsenite and cadmium sensitivity are the same in *hmt2*Δ cells, and therefore if free cadmium prevents up-regulation of glutathione synthesis (Vande Weghe and Ow 2001), then free arsenic likely also has the same effect.

### Hmt2 electron acceptor dependency likely explains CoQ_10_ biosynthesis requirement in As/Cd resistance

As the name implies, sulfide-quinone oxidoreductase uses quinone as an electron acceptor when converting sulfide into sulfur. This requirement most likely explains why our cadmium and arsenic sensitivity screens identified five components of the ubiquinone/coenzyme Q_10_ (CoQ_10_) biosynthesis pathway: Coq3, Coq4, Coq5, Coq9, and Dps1 (Figure 5). As discussed below, four additional components of the CoQ_10_ biosynthesis pathway were identified in the cadmium (Coq10) and arsenite screens (Abc1, Coq7, Coq11). Four of these genes (Coq3, Coq 4, Coq 5, and Coq 7) and another CoQ_10_ biosynthesis component (Coq2) were also identified in our previous cadmium screen (Kennedy *et al.* 2008), which collectively account for most of the proteins of the CoQ_10_ biosynthesis pathway (Figure 5). We further note that as observed with *hmt2*Δ cells, most of the CoQ_10_ biosynthesis mutants displayed high expressivity for both cadmium and arsenite sensitivity, that is, they were among the most sensitive mutants (Table S3 and Table S4). These results suggest that Hmt2 sulfide-quinone oxidoreductase activity is critical for tolerance of both cadmium and arsenite and this activity absolutely depends on biosynthesis of CoQ_10_ to act as an electron donor during sulfide oxidation.

### Mitochondrial translation elongation, pyruvate transport, and oxidative phosphorylation are required for As/Cd resistance

The CoQ_10_ biosynthesis pathway, the sulfide-quinone oxidoreductase Hmt2, and key proteins involved in cysteine biosynthesis such as cysteine synthase Cys11 and serine *O*-acetyltransferase Cys2 are found in the mitochondria. It is thus interesting that additional proteins required for As/Cd resistance have been localized to the mitochondria. Notably, the predicted mitochondrial translation elongation factor G encoded by *mef2* was found to be critical for As/Cd resistance (Table 2). SPBC660.10 is orthologous to MEF2 in *S. cerevisiae* and GMF2 in humans. These mitochondrial GTPases mediate the disassembly of ribosomes from messenger RNA at the termination of mitochondrial protein biosynthesis. Studies in budding yeast indicate that inactivation of MEF2 results in loss of mitochondrial DNA, defective oxidative phosphorylation, and reduced chronological lifespan (Callegari *et al.* 2011). Oxidation of active cysteine groups in mitochondrial proteins not only helps to maintain redox homeostasis but also plays a critical role in oxidative stress signaling (Bak and Weerapana 2015). Interestingly, we observed that *mef2*Δ mutants grew poorly in EMM2 media and this defect was partially suppressed by addition of cysteine but not methionine (Figure S2). Thus in *S. pombe*, which is similar to humans in mitochondrial physiology (Chiron *et al.* 2005), *mef2*Δ mutants could be expected to have a heightened requirement for cysteine for maintenance of general mitochondrial function and cell survival, especially in the presence of cadmium or arsenite.

Our screens also revealed that the mitochondrial NADH:ubiquinone oxidoreductase encoded by *nde2* is critical for As/Cd resistance (Table 2). This enzyme is essential for mitochondrial oxidative phosphorylation (Hirst 2013). Both Nde1 and Nde2 encode NADH:ubiquinone oxidoreductase proteins in fission yeast, suggesting that a partial loss of enzyme activity causes As/Cd sensitivity while maintaining cell viability. Interestingly, as the name implies, NADH:ubiquinone oxidoreductase uses CoQ_10_ as an electron acceptor, thus its important function in As/Cd resistance highlights the requirement for proteins of the ubiquinone biosynthesis pathway as described above (Figure 5).

The importance of mitochondrial oxidative phosphorylation for As/Cd resistance was also indicated by the identification of mitochondrial pyruvate transmembrane transporter Mpc2 in our screen. Transport of pyruvate into the mitochondria is important because pyruvate oxidation is required for efficient ATP production (McCommis and Finck 2015). Mpc1 and Mpc2 share responsibilities for mitochondrial pyruvate transmembrane transport in fission yeast, suggesting that cells lacking Mpc2 suffer a partial defect in mitochondrial oxidative phosphorylation that results in As/Cd sensitivity. Human MPC1 is deleted or underexpressed in multiple cancer types and correlates with poor prognosis; furthermore, re-expression of MPC1 or MPC2 in cancer cells impaired anchorage-independent growth (Schell *et al.* 2014).

### Requirement for pyridoxal 5′-phosphate (vitamin B6) in As/Cd resistance

**Figure 6:**
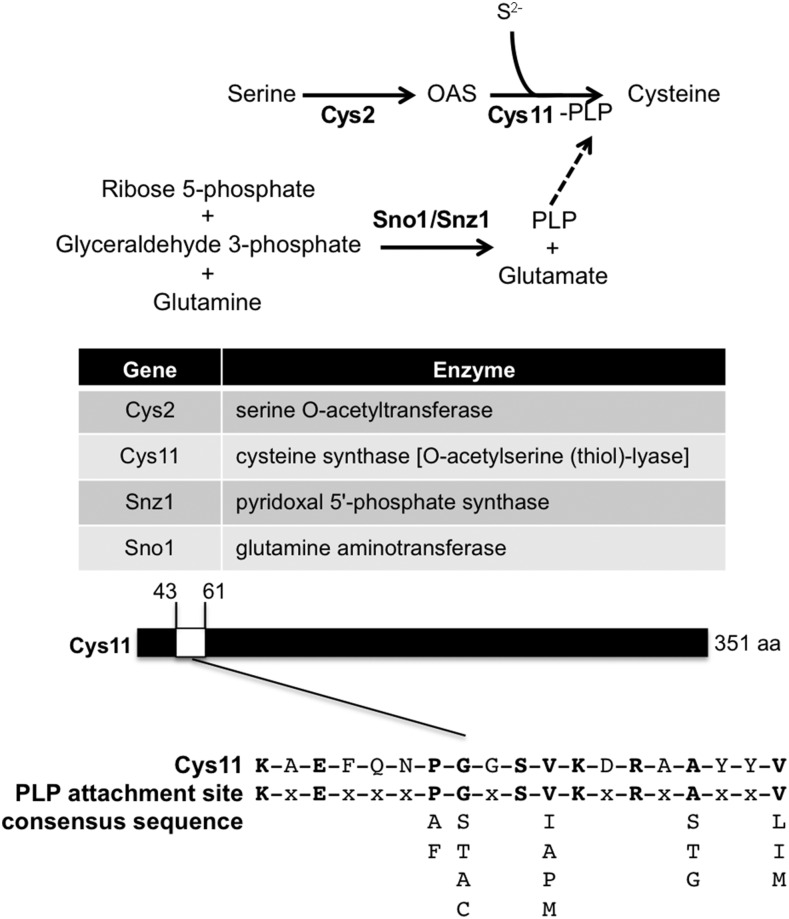
Requirement for pyridoxal 5′-phosphate in As/Cd resistance. PLP is a coenzyme for cystathionine β-synthase, which converts serine and homocysteine into cystathionine. Snz1 is essential for PLP synthesis with a putative role as a subunit of PLP synthase, which can explain why our screens revealed that Snz1 is required for cadmium and arsenic tolerance. Consistent with the proposal that Cys11 is a cystathionine β-synthase, we found that it has a predicted PLP attachment site typical of cysteine synthase/cystathionine β-synthase enzymes. Sno1/SPAC222.08c encodes a predicted glutamine aminotransferase which is likely also required for PLP synthesis, thus explaining why Sno1 is required for arsenic resistance and probably also important for cadmium tolerance.

Our functional profiling screens found that Snz1 is required for cadmium and arsenite tolerance (Table S5). Genetic studies revealed that *snz1*Δ cells are pyridoxine auxotrophs, confirming that Snz1 is essential for pyridoxal 5′-phosphate (PLP) synthesis (Stolz *et al.* 2005) with a putative role as a subunit of PLP synthase. PLP is the active form of vitamin B6, which is a coenzyme in a variety of enzymatic reactions (Eliot and Kirsch 2004). Notably, PLP is a coenzyme for cystathionine β-synthase, which converts serine and homocysteine into cystathionine, which subsequently is transformed into cysteine by cystathionine γ-lyase (Selhub 1999). However, fission yeast lacks cystathionine β-synthase and cystathionine γ-lyase (Brzywczy *et al.* 2002). *S. pombe* instead relies on cysteine synthase [*O*-acetylserine (thiol)-lyase] to catalyze synthesis of cysteine from O_3_-acetyl-L-serine and hydrogen sulfide (Figure 2). We note that cysteine synthase Cys11 in fission yeast has a predicted PLP attachment site typical of cysteine synthase/cystathionine β-synthase enzymes (Saito *et al.* 1993; Swaroop *et al.* 1992) (Figure 6). Therefore it is likely that Snz1 was identified in our screen because cysteine synthase Cys11 is a PLP-stimulated enzyme. Further, in the deoxyxylulose-5-phosphate (DOXP) independent pathway for PLP synthesis, PLP synthase acts with glutamine aminotransferase to synthesize PLP from D-ribose 5-phosphate + D-glyceraldehyde 3-phosphate + L-glutamine (Mukherjee *et al.* 2011). It is noteworthy that our arsenite screens uncovered glutamine aminotransferase Sno1/ SPAC222.08c as well as Snz1 (Table S4). In fact, our cadmium screens suggested that Sno1 might also be required for cadmium resistance, as the *sno1*Δ mutant ranked 135, 290, 1367, and 269 among ∼3000 mutants in the four cadmium sensitivity screens (Table S2). Taken together, these findings show that vitamin B6 biosynthesis is critical for cadmium and arsenite tolerance in fission yeast.

### Relationship between oxidative stress and As/Cd sensitivity

Heavy metals like arsenic and cadmium have been implicated in inducing oxidative stress. However, the mechanism of oxidative stress generated by As/Cd remains largely unexplored. Free cadmium and arsenic can cause oxidative stress, either through depletion of intracellular glutathione, inhibition of antioxidant enzymes (*i.e.*, superoxide dismutase, peroxidase, and catalase), or displacement of redox-active transition metals such as iron and copper from proteins. Any of these effects will increase ROS and consequently intracellular oxidative stress (Cuypers *et al.* 2010; Hughes 2002; Stohs and Bagchi 1995; Valko *et al.* 2005). As noted above, AnGeLi identified “sensitive to hydrogen peroxide” as one of the annotated mutant phenotypes that were most highly enriched among the 36 genes required for arsenite and cadmium tolerance (Table 3). For example, sulfate adenyltransferase Sua1 and cysteine synthase Cys11 were found in our As/Cd screens and in a screen for mutants that are unable to grow on H_2_O_2_-containing fermentable media (Zuin *et al.* 2008). The thiol-peptide glutathione (GSH) not only serves as the precursor to phytochelatin biosynthesis, but also acts as the primary antioxidant inside cells that prevents proteins from undergoing irreversible oxidative damage. Hence it is not surprising that genes involved in the sulfate assimilation pathway leading to glutathione biosynthesis are required for tolerance of cadmium and arsenite as well as for tolerance of oxidative stress caused by hydrogen peroxide. However, the question arises as to whether we identified these genes in our As/Cd screens because they are required to synthesize glutathione as a precursor of the metal chelator phytochelatin, or were they identified because the antioxidant property of glutathione is required to neutralize ROS generated by arsenic and cadmium. The mutant lacking phytochelatin synthetase Pcs2 is probably most informative on this question. The *pcs2*Δ mutant is acutely sensitive to both arsenite and cadmium sulfate and yet it was not found in the hydrogen peroxide screen nor was it sensitive to H_2_O_2_ when previously tested (Kennedy *et al.* 2008; Zuin *et al.* 2008). On the other hand, previous studies showed the transcription factor Prr1 is especially critical for H_2_O_2_-tolerance (Ohmiya *et al.* 1999), whereas in our assays the *prr1*Δ mutant was insensitive to cadmium and arsenite (Table S2). The same relationship applies to mutants defective in cytochrome c oxidase subunit VI Cox6 (Zuin *et al.* 2008), mitochondrial respiratory chain complex IV assembly protein Cox19 (Zuin *et al.* 2008), Ctt1 catalase (Mutoh *et al.* 1999; Paulo *et al.* 2014), and ubiquinol-cytochrome-c reductase complex subunit 5 Rip1 (Zuin *et al.* 2008). All of these mutants are acutely sensitive to hydrogen peroxide but were insensitive to either cadmium or arsenite in our assays (Table S2). These observations suggest that while our As/Cd screens enriched mutants that are sensitive to hydrogen peroxide, their role in phytochelatin synthesis leading to metal sequestering and detoxification is the primary reason for their observed sensitivity as opposed to providing tolerance from metal-induced oxidative stress.

### Genes specifically required for arsenite resistance

The functional profiling screens identified 110 genes that are required for arsenite resistance, of which some of the most important as judged by GI scores are involved in sulfate assimilation leading to cysteine and glutathione biosynthesis (*cys2*, *sua1*, *met14*, *gcs2*), siroheme biosynthesis (*met8*), and CoQ_10_ biosynthesis (*coq9*, *coq5*, *dps1*) (Table S4). As discussed above these are among the 36 genes that are also required for cadmium resistance (Table S5). Here we comment on the 74 genes that were identified as being required for arsenite but not cadmium tolerance (Table S4).

### Prefoldin-mediated assembly of unfolded proteins is important for arsenite resistance

AnGeLi found that the Biological Process “tubulin complex assembly” and the Molecular Function “unfolded protein binding” were among enriched GO terms in the arsenite-specific list of genes (Table 4). These lists were mostly overlapping and largely consisted of the prefoldin subunits; indeed, “prefoldin complex” was highly enriched among the GO cellular component terms (Table 4). In fact, all subunits of the heterohexameric prefoldin protein complex (Gim6, Gim4, Pac10, Gim3, Bob1, and Gim1) were identified in the arsenite screen. These findings are interesting in light of recent studies suggesting that unfolded proteins are targets of heavy metals (Tamas *et al.* 2014). These studies indicate that heavy metals and metalloids, particularly arsenite, can inhibit refolding of denatured proteins *in vitro*, are able to disrupt protein folding *in vivo*, and cause nascent protein aggregation in cells (Jacobson *et al.* 2012; Ramadan *et al.* 2009; Sharma *et al.* 2008). Misfolded proteins are cytotoxic because they tend to aggregate or interact inappropriately with other proteins, and it is noteworthy that protein misfolding and aggregation have been linked to many diseases (Hartl *et al.* 2011; Powers *et al.* 2009). Furthermore, there is increasing evidence that heavy metals can promote the aggregation of disease-associated proteins, and altered metal homeostasis may enhance the progression of neurodegenerative diseases (Caudle *et al.* 2012; Savelieff *et al.* 2013).

**Table 4:** Summary of GO categories enriched by mutants sensitive to arsenite but not cadmium (background is protein-encoding genes)

GO Category	Term	List Frequency (74 total)	Background Frequency (5135 total)	p Value	Genes
**Biological process**	Protein targeting to peroxisome	6.76% (5)	0.21% (11)	3.67e−05	*pex1*, *pex5*, *pex12*, *pex13*, *pex19*
	Tubulin complex assembly	8.11% (6)	0.39% (20)	3.22e−05	*bob1*, *gim1*, *gim3*, *gim4*, *gim6*, *pac10*
	Mitochondrial transport	10.81% (8)	1.85% (95)	0.00551	*SPAC823.10c*, *SPBC336.^13^C*, *atp1*, *atp14*, *atp2*, *hot15*, *tom7*, *tom70*
**Molecular function**	Unfolded protein binding	9.46% (7)	0.84% (43)	0.00263	*atp10*, *bob1*, *gim1*, *gim3*, *gim4*, *gim6*, *psh3*
**Cellular component**	Prefoldin complex	8.11% (6)	0.12% (6)	5.82e−09	*bob1*, *gim1*, *gim3*, *gim4*, *gim6*, *pac10*
	Mitochondrion	39.19% (29)	14.10% (724)	1.89e−05	*SPAC1071.11*, *SPAC1486.01*, *SPAC823.10c*, *SPBC106.07c*, *SPBC336.^13^C*, *SPBC365.16*, *SPBC3H7.03c*, *abc1*, *atp1*, *atp10*, *atp11*, *atp14*, *atp2*, *cit1*, *coq11*, *coq7*, *eca39*, *hot15*, *ilv1*, *mdh1*, *mrpl1*, *mss116*, *pos5*, *ppr6*, *ppr7*, *qcr9*, *tom7*, *tom70*, *trx2*
	Peroxisome	6.76% (5)	0.47% (24)	0.00293	*pex1*, *pex5*, *pex12*, *pex13*, *pex19*

These findings correlate well with a genome-wide screen of haploid deletion mutants in budding yeast, which found that strains lacking subunits of GimC/prefoldin protein complex were among the most arsenite-sensitive mutants (Pan *et al.* 2010). This study further found that heterozygous diploid deletion mutants of the essential chaperonin complex TRiC were also very sensitive to arsenite; moreover, arsenite inhibited the ability of purified TRiC to promote refolding of chemically denatured actin *in vitro*. Coupled with evidence of synthetic negative genetic interactions among mutations impairing TRiC and GimC (Siegers *et al.* 1999), these studies suggested that arsenite directly inhibits TRiC, which works with prefoldin to ensure the proper folding and oligomerization of actin, α-tubulin and β-tubulin (Pan *et al.* 2010). In contrast to investigations in mammalian cells (Zhang *et al.* 2007), studies in budding yeast did not support the idea that arsenite blocks microtubule polymerization by directly binding β-tubulin (Pan *et al.* 2010). We note that as in *S. cerevisiae*, all subunits of TRiC are essential for cell viability in fission yeast, and thus TRiC genes were not part of our haploid deletion screens (Hayles *et al.* 2013; Kim *et al.* 2010). We also note that in contrast to the study in budding yeast in which strains lacking GimC/prefoldin genes were some of the most arsenite-sensitive mutants, in fission yeast we found that these mutants displayed low to medium expressivity in the arsenite screen.

### Protein targeting to the peroxisome and mitochondrial transport are required for arsenite resistance: a peroxisome–mitochondrion connection?

AnGeLi uncovered “protein targeting to peroxisome” as a highly enriched GO biological process term among the genes that are specifically required for arsenite resistance (Table 4). Included in this list is Pex5, which binds to the C-terminal PTS1-type tripeptide peroxisomal targeting signal (SKL-type) and plays an essential role in peroxisomal protein import (Hettema *et al.* 2014; Platta and Erdmann 2007). Peroxisome functions can vary somewhat between organisms but generally it plays an important role in energy metabolism, notably involving β oxidation of fatty acids. These reactions produce hydrogen peroxide that is then detoxified by catalase to prevent oxidative stress. Arsenic has the potential to cause oxidative stress but deletion of *ctt1* encoding the sole catalase in fission yeast did not cause arsenite sensitivity (Table S2), even though this mutant is quite sensitive to exogenous hydrogen peroxide (Mutoh *et al.* 1999; Paulo *et al.* 2014).

Mitochondrial behavior is governed by interactions with other organelles (Klecker *et al.* 2014), including peroxisomes in fission yeast and budding yeast (Jourdain *et al.* 2008; Neuspiel *et al.* 2008). As mitochondrial functions are critical for arsenite resistance (see above and Table 4), one possibility is that defects in protein targeting to the peroxisome might impair aspects of mitochondrial function that are important for arsenite resistance. This possibility gains further support from the observation that “mitochondrial transport” was a significantly enriched GO biological process term among the proteins specifically required for arsenite resistance (Table 4). These proteins include subunits of the F1-ATPse complex (Atp1, Atp2, Atp14), as well as subunits of Translocase of Outer Membrane (Tom 7 and Tom70) and Translocase of Inner Membrane (Hot15).

### Mitochondrial thioredoxin Trx2 is required for arsenite resistance

Two oxidoreductase enzymes, thioredoxin and thioredoxin reductase, act with the glutathione system to maintain reductive intracellular redox potential. Thioredoxin is a small protein (12–15 kDa) that directly binds trivalent arsenic, likely through the highly conserved Cys-Gly-Pro-Cys motif (Shen *et al.* 2013; Wang *et al.* 2007). Thioredoxin could be expected to be important for arsenic resistance in eukaryotes but genetic evidence has been lacking. As seen in most eukaryotes, fission yeast has distinct cytosolic and mitochondrial thioredoxins. Our screens revealed that the mitochondrial thioredoxin Trx2 is required for arsenic resistance in fission yeast (Table S4). The *trx2*Δ mutant was insensitive to cadmium in our functional profiling screen, although we note it displayed weak sensitivity to cadmium in our previous screen carried out on solid media (Kennedy *et al.* 2008). The strain lacking the cytosolic thioredoxin Trx1 was absent from our deletion library.

### Oxoglutarate dehydrogenase complex, an arsenite target in humans, is critical for arsenite resistance in fission yeast

The gene *kgd1/SPBC3H7.03c* was identified as being essential for arsenite resistance (Table S4). This gene encodes the predicted E1 component of 2-oxoglutarate dehydrogenase complex (OGDC), also known as α-ketoglutarate dehydrogenase (KGDH) complex, which catalyzes the reaction: α-ketoglutarate + NAD^+^ + CoA → succinyl CoA + CO_2_ + NADH. This reaction is a key step of the tricarboxylic acid (TCA) cycle required for aerobic respiration and energy production. Lipoic acid (lipoamide), an organosulfur compound, is an essential OGDC cofactor that interconverts into dihydrolipoic acid (dihydrolipoamide). As(III) reacts with the thiol groups of dihydrolipoamide, thereby inhibiting OGDC and potentially other lipoamide-dependent enzymes such as pyruvate dehydrogenase (PDH) that is also required for the TCA cycle (Hughes 2002; Shen *et al.* 2013). Indeed, inhibition of OGDC and PDH are thought to underlie much of arsenite toxicity in humans. Aspartate aminotransferase Aat2/SPAC10F6.^13^C, a PLP-dependent enzyme that is responsible for providing α-ketoglutarate for OGDC, was also highlighted in our arsenite screen (Table S4).

Interestingly, our screens also identified the TCA cycle enzymes citrate synthase Cit1 and malate dehydrogenase Mdh1 as being important for arsenite resistance (Table S4).

### DNA damage checkpoint and double-strand break repair mutants are insensitive to arsenite

Arsenic has carcinogenic properties yet it remains unclear whether it causes genome instability, and if so, whether arsenic directly or indirectly creates DNA damage. A recent study with *S. cerevisiae* indicated that arsenite causes replication and transcription-independent double-strand breaks (DSBs) in all phases of the cell cycle, triggering DNA damage checkpoint responses and homology dependent repair (HDR) requiring members of the Rad52 epistasis group (Litwin *et al.* 2013). These studies further indicated that arsenite has similar effects in fission yeast. However, our arsenite screen did not reveal any role for core DSB repair or checkpoint proteins in arsenite resistance (Table S4). These proteins include subunits of the Rad52 epistasis group (Rad52, Rad54, Rad55, Rad57) required for HDR, the Mre11 epistasis group (Mre11^Rad32^, Ctp1) that detects and initiates nucleolytic processing of DSBs for HDR, Mus81-Eme1 Holliday Junction resolvase (Mus81) that in mitotic cells is specifically required for recovery from replication-induced DSBs, and subunits of the Rad3^ATR^ epistasis group (Rad26^ATRIP^, Rad1, Rad9, Hus1, Rad17, Crb2) that is required for the DSB-induced cell cycle checkpoint (Langerak and Russell 2011). Members of the Cds1^Chk2^ epistasis group (Cds1, Mrc1) that are specifically required for survival of replication fork arrest were also insensitive to arsenite in our screen. Most of these genes were identified in a similar functional profiling screen carried out with well-established DNA damaging agents and DNA replication inhibitors (Han *et al.* 2010). Thus arsenite concentrations that modestly inhibit growth of wild-type cells do not cause levels of DNA damage that will lead to cell death in the absence of core DNA damage responses. However, we note that the arsenite concentrations used in our screen (100–200 μΜ NaAsO_2_) were much lower than those used by Litwin and colleagues (500–4000 μΜ NaAsO_2_) in their studies with *S. cerevisiae* and *S. pombe* (Litwin *et al.* 2013). Therefore, it appears that DNA damage responses can be important for coping with very high concentrations of arsenite in fission yeast, or tolerating simultaneous exposure to arsenite and other toxins, but at lower arsenite concentrations other cellular activities are much more important, particularly those involved in arsenite detoxification. In this respect *S. cerevisiae* and *S. pombe* appear to be similar, as DNA damage responses were not highlighted in corresponding arsenite functional profiling screens of budding yeast (Thorsen *et al.* 2009). With respect to carcinogenesis, our studies do not exclude the possibility that arsenite increases mutagenic lesions that are not generally toxic in the absence of core DNA damage responses, or that arsenite might inhibit genome protection mechanisms and thereby increase mutagenesis caused by other internal or external DNA damaging agents. On this point it is interesting that arsenite synergistically increased genotoxicity of phleomycin but not other DNA damaging agents in budding yeast (Litwin *et al.* 2013).

The arsenite screen revealed that cells lacking Spd1 are sensitive to arsenite (Table S4). Spd1 is a stoichiometric inhibitor of ribonucleotide reductase (RNR), which is the key rate-limiting activity required for synthesis of deoxyribonucleotide triphosphates (dNTPs) (Hakansson *et al.* 2006). RNR provides the cell with a balanced supply of dNTPs for DNA replication and repair. RNR activity depends on thioredoxin acting as an electron donor. Thioredoxin also directly binds trivalent arsenic as mentioned above. It is therefore tempting to speculate that elimination of the RNR inhibitor Spd1 coupled with arsenic binding to thioredoxin leads to RNR malfunction, which perhaps leads to genome instability.

Our arsenite screen also uncovered Ddb1 as being important for arsenite resistance. Ddb1 is a subunit of the Pcu4-Ddb1-CSN ubiquitin ligase that is required for resistance to some drugs that interfere with DNA replication (Liu *et al.* 2005). This activity involves degradation of Spd1. However, Ddb1 is also required for resistance to antifungal agents that do not damage DNA or inhibit replication (Fang *et al.* 2012), which it shares with a subset of other proteins identified in our screen that have no involvement in DNA damage responses.

### Cadmium-specific resistance genes

The functional profiling screens identified 106 genes that are required for cadmium resistance, of which those involved in sulfate assimilation leading to glutathione and phytochelatin biosynthesis were among the most important as judged by GI scores (Table S3). Of these 106 genes, 70 were identified as being required for cadmium but not arsenite tolerance. Some of these genes have well-characterized roles in cadmium resistance. For example, the transcription factor Zip1 plays a critical role in the cadmium-induced transcriptional induction of a subset of genes that is critically required for cadmium resistance (Harrison *et al.* 2005). Notably and as discussed above, the uroporphyrin-3 *C*-methyltransferase gene SPCC1739.06c required for siroheme biosynthesis, the Cys2 gene encoding a serine *O*-acetyltransferase required for cysteine biosynthesis, and the mitochondrial sulfide-quinone oxidoreductase Hmt2 required to prevent accumulation of toxic levels of sulfide during cadmium stress are all transcriptionally up-regulated via a Zip1-dependent mechanism in response to cadmium exposure (Harrison *et al.* 2005). Here we shall consider additional functional classes of genes identified in our screen.

### SAGA transcriptional coactivator controls expression of genes required for cadmium resistance

AnGeLi identified the evolutionarily conserved “SAGA complex” as a highly enriched GO cellular component term among the genes specifically required for cadmium resistance (Table 5). The SAGA complex (Spt–Ada–Gcn5 acetyltransferase) is a multifunctional coactivator of transcription, which is best known for its histone H3 acetyltransferase (HAT) activity provided by a functional module consisting of Gcn5, Ada2, and Ngg1/Ada1 (Helmlinger *et al.* 2008). Accordingly, the six identified SAGA mutants (*ada2*, *gcn5*, *ngg1*, *sgf29*, *sgf73*, and *tra1*) composed the most enriched GO biological process term of “histone acetylation” (Table 5). Indeed, strains lacking Gcn5, Ngg1, or Tra1 were among the most cadmium-sensitive mutants (Table S3). Two additional SAGA genes, *spt20* and *sgf11*, were identified among the 36 genes required for both arsenic and cadmium resistance (Table S5), totaling 8 of 19 reported subunits of the SAGA complex (Helmlinger *et al.* 2008), and accounting for the very high enrichment of the GO cellular component term “SAGA complex” (p value = 5.83e−07) among all 106 genes identified in the cadmium screen.

**Table 5:** Summary of GO categories enriched by mutants sensitive to cadmium but not arsenic (background is protein-encoding genes)

GO Category	Term	List Frequency (74 total)	Background Frequency (5135 total)	p Value	Genes
**Biological process**	Vesicle-mediated transport	24.29% (17)	6.27% (322)	0.000486	*akr1*, *apl5*, *apm3*, *bhd1*, *cfp1*, *dop1*, *fsv1*, *imh1*, *pof6*, *sat1*, *syj1*, *tlg2*, *vps17*, *vps26*, *vps29*, *vps35*, *vs.l1*
	Post-Golgi vesicle-mediated transport	10.00% (7)	0.82% (42)	0.000617	*apl5*, *apm3*, *bhd1*, *cfp1*, *dop1*, *fsv1*, *vs.l1*
	Histone acetylation	8.57% (6)	0.90% (46)	0.00815	*ada2*, *gcn5*, *ngg1*, *sgf29*, *sgf73*, *tra1*
**Cellular component**	SAGA complex	8.57% (6)	0.37% (19)	6.87e−05	*ada2*, *gcn5*, *ngg1*, *sgf29*, *sgf73*, *tra1*
	Retromer complex	5.71% (4)	0.10% (5)	7.15e−05	*vps17*, *vps26*, *vps29*, *vps35*
	Endosome	11.43% (8)	1.56% (80)	0.00254	*akr1*, *aut12*, *fsv1*, *tlg2*, *vps26*, *vps29*, *vps35*, *vs.l1*

**Figure 7:**
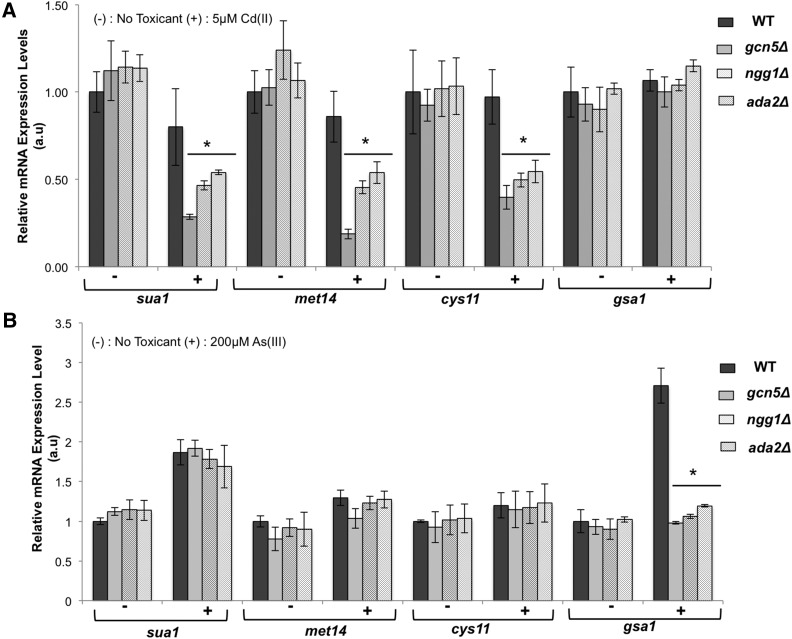
SAGA modulates expression of genes in the sulfate assimilation pathway following cadmium stress. mRNA expression analysis of genes involved in cysteine (*sua1*, *met14*, and *cys11*) and glutathione (gsa1) biosynthesis by RT-PCR reveals a critical requirement for SAGA-HAT module in maintaining full expression of these genes following cadmium exposure (A) but is not essential for tolerating arsenite stress (B). Asterisks denote statistically significant differences between wild-type and SAGA mutants as determined by two-tailed Student’s *t*-test, 0.002 ≤ p ≤ 0.02. All data were normalized with *act1* mRNA as the internal control and mRNA from wild type without toxin treatment as the calibrator. Only changes >1.5-fold were considered significant. Error bars represent SD values computed based on at least three technical repeats.

SAGA modulates gene expression in response to nitrogen limitation and oxidative stress (Helmlinger *et al.* 2008; Sanso *et al.* 2011), and SAGA mutants were found in a previous screen of cadmium-sensitive mutants (Kennedy *et al.* 2008), but it is unclear if SAGA controls expression of genes that are required for cadmium resistance. To address this question we used real-time PCR to measure expression of the genes encoding sulfate adenyltransferase Sua1, adenylyl-sulfate kinase Met14, and cysteine synthase Cys11. As reported above, these genes are required for cysteine biosynthesis and are critical for cadmium tolerance. Expression of *sua1*, *met14*, and *cys11* was largely unaffected by 5 μM cadmium sulfate exposure in wild type (Figure 7), as previously observed with 500 μM CdSO_4_ (Chen *et al.* 2003). Loss of the HAT-specific SAGA subunits Gcn5, Ngg1, or Ada2 did not impact *sua1*, *met14*, or *cys11* expression in the absence of cadmium stress, in accordance with previous microarray data (Helmlinger *et al.* 2011). However, in the presence of 5 μM CdSO_4_ the SAGA mutants were unable to maintain expression of these cysteine biosynthesis genes (Figure 7A). In the most extreme case *met14* mRNA was reduced ∼fourfold in *gcn5*Δ cells relative to wild type. However, this pattern was not uniform for all genes encoding enzymes leading to glutathione biosynthesis. Notably, irrespective of cadmium exposure expression of the *gsa1* gene, encoding glutathione synthetase required for cadmium resistance, was largely unaffected by elimination of the HAT subunits of SAGA (Figure 7A). We also examined the expression of *gst2* encoding glutathione *S*-transferase, which is strongly up-regulated following cadmium exposure even though it does not appear to be required for cadmium resistance (Chen *et al.* 2003). The level of *gst2* mRNA decreased in both pre- and postcadmium exposure conditions for SAGA deletion mutants (Figure S3). Taken together these results suggest that while SAGA is not generally required for transcriptional up-regulation in response to cadmium stress it is important for full expression of genes that are critical for cadmium resistance, which could plausibly account for the cadmium sensitivity of SAGA mutants.

We note that while cysteine biosynthesis is critical for arsenite resistance, the SAGA complex was not an enriched GO term among the arsenite-sensitive mutants. To address this issue we measured *sua1*, *met14*, and *cys11* expression following exposure to 200 μM arsenite and found that loss of the aforementioned SAGA subunits did not significantly impact expression of these genes (Figure 7B). An arsenite-induced increase in *gsa1* expression was ablated in the SAGA mutants but this defect must not have caused sufficient arsenite sensitivity to be detected in our screen (Figure 7B). We further note that mutants lacking three SAGA subunits (Sgf11, Spt8, and Spt20) were identified in the arsenite screen but these mutants had low expressivity.

### Plasma membrane transporters and vacuolar transporters required for cadmium resistance

AnGeLi identified the GO biological process terms and “vesicle-mediated transport” and more specifically “post-Golgi vesicle-mediated transport” as being significantly enriched among the 70 mutants that were specifically sensitive to cadmium, along with the GO cellular component terms “retromer complex” and “Endosome” (Table 5). These terms encompass overlapping sets of genes that collectively account for 17/70 of the cadmium-specific sensitive mutants. Of particular interest are the genes encoding four of the five subunits of the retromer complex (Vps17, Vps26, Vps29, and Vps35) involved in vacuolar protein sorting and another group of proteins involved in Golgi to vacuole vesicle-mediated transport (Apl5, Apm3, Fsv1, and Vsl1) (Takegawa *et al.* 2003). Thus the vacuolar degradation pathway appears to play a crucial role in cadmium detoxification.

Our screen also identified the vacuolar transmembrane transporter Hmt1 as a protein that is required for cadmium tolerance, in agreement with previous studies (Ortiz *et al.* 1992; Sooksa-Nguan *et al.* 2009). Hmt1 is thought to transport Cd–PC complexes into the vacuole, but more recent studies established that Hmt1 is able to confer Cd resistance by a pathway that is at least partially independent of phytochelatin (Mendoza-Cozatl *et al.* 2010; Preveral *et al.* 2009; Sooksa-Nguan *et al.* 2009). This PC-independent function of Hmt1 may explain why it was highlighted as being critical for tolerance of cadmium but not arsenite.

The cadmium screen also identified a number of genes that are directly involved in intracellular transport of metals. Since most metals enter the cell by mimicking other essential cations like Ca^2+^ or Zn^2+^, cells have also adapted the use of several facilitated divalent cation transporters to recognize and aid in the efflux of heavy metals. Mfc1, which contains a (MFS) major facilitator superfamily domain, is reported to be a copper transmembrane transporter in meiotic and sporulating cells (Beaudoin *et al.* 2011). Our screen revealed that Mfc1 has a crucial role in mediating cadmium resistance in mitotic cells grown in liquid media (Table S3). The calcium-transporting P-type ATPase Pmr1 was also found to be important for cadmium resistance (Table S3), as previously reported (Cortes *et al.* 2004; Kennedy *et al.* 2008). Our screen revealed that regulation of calcium ion import across the plasma membrane by Cch1 is also critical for cadmium resistance (Table S3) (Ma *et al.* 2011).

### Comparisons to *S. cerevisiae*

In this study we have reported the outcome of functional profiling screens to identify genes in the fission yeast haploid deletome that are most critical for tolerance of arsenite and cadmium. Thorsen and colleagues, who identified 306 arsenite-sensitive and 382-cadmium-sensitive mutants in budding yeast, with an overlap of 106 mutants, performed the most recent and directly comparable screens of *S. cerevisiae* (Thorsen *et al.* 2009). GO term enrichment of the 106 genes required for resistance to both metals in budding yeast highlighted key terms such as “sulfur compound biosynthetic process” (ninefold enrichment, p value = 2.40e−04), “late endosome to vacuole transport” (14-fold, 3.87e−08), and terms related to transcriptional regulation, for example “positive regulation of transcription from RNA polymerase II promoter” (fourfold, 1.50 e−03). Of these GO terms only “sulfur compound biosynthetic process” (21-fold enrichment, p value = 7.01e−07) was shared with the corresponding list of 36 genes we reported for fission yeast. Additional GO terms enriched in the *S. pombe* list but not in *S. cerevisiae* list include “ubiquinone biosynthetic process” (56-fold, 6.86e−06), “glutathione biosynthetic process” (104-fold, 2.70e−04), and “siroheme biosynthetic process” (139-fold, 4.64e−03). In general the GO terms identified in fission yeast were more specific and highly enriched, which might reflect differences with the annotation databases arising in part from the smaller and less redundant genome of *S. pombe*. For example, glutathione biosynthesis is certainly critical for arsenite and cadmium resistance in *S. cerevisiae*, indeed *GCS1* encoding glutamate-cysteine ligase was found to be required for arsenite and cadmium resistance (Thorsen *et al.* 2009), yet the expected GO term was not enriched. Similarly, although GO terms associated with ubiquinone and siroheme biosynthesis were not enriched in the budding yeast screens, it seems probable that these pathways are important for arsenite and cadmium resistance in *S. cerevisiae*, given that these cofactors are critical for the activity of key enzymes involved in cysteine biosynthesis. Other expected GO terms enriched with fission yeast such as “cellular response to metal ion” (44-fold, 2.70e−04) and “cellular response to cadmium ion” (69-fold, 9.09e−04) were not significantly enriched in budding yeast. On the other hand, we note that orthologous stress-activated protein kinases, *S. cerevisiae* Hog1 and *S. pombe* Sty1/Spc1, were identified as being required for arsenite and cadmium resistance in the two yeasts.

Thorsen and colleagues also compiled “core-sets” of genes that were identified in at least two of the cadmium or arsenic screens that have been reported for *S. cerevisiae* (Thorsen *et al.* 2009). These lists comprised 89 As-sensitive and 209 Cd-sensitive mutants, with an overlap of 31 genes. GO term analysis of this list identified a different list of enriched terms, notably “positive regulation of transcription by galactose” (97-fold, 1.52e−03) and “response to metal ion” (26-fold, 4.08e−03). GO terms associated with sulfur compound or glutathione biosynthesis, intracellular transport, or enzyme cofactor biosynthesis were not enriched among these 31 genes. From these comparisons one could draw the conclusion that arsenic and cadmium defense mechanisms are largely nonoverlapping in budding yeast, but we believe this lack of concordance reflects experimental variations that confound analyses even when considering only “core-sets” of genes. That said, it does appear that even when only considering results from screens that were performed in parallel (Thorsen *et al.* 2009), the data from budding yeast screens suggest a lower degree of overlap between arsenic and cadmium tolerance mechanisms, as compared to fission yeast.

Thorsen and colleagues identified 200 genes that were required for arsenite but not cadmium resistance in budding yeast (Thorsen *et al.* 2009). Notable GO terms that were enriched among these genes included “tubulin complex assembly” (40-fold enrichment, p value = 1.48e−08) and “mitochondrial translation” (fivefold, 2.80e−07). Our comparable list of 74 arsenite-specific genes also enriched “tubulin complex assembly” (35-fold enrichment, 1.10e−05) as well as peroxisome-related terms such as “protein targeting to peroxisome” (32-fold, 1.36e−04). As noted above, the six fission yeast genes identified under the GO term “tubulin complex assembly” account for all the subunits of the heterohexameric prefoldin protein complex. Remarkably, these genes are the orthologs of the six genes comprising the “tubulin complex assembly” GO term list identified in *S. cerevisiae* (Thorsen *et al.* 2009). This correlation points to the critical and evolutionarily conserved role of prefoldin in combatting the toxic effects of arsenic. As mentioned above, inhibition of protein folding and promotion of protein aggregation is an emerging theme of heavy metal toxicity and could underlie some neurodegenerative and age-related syndromes (Caudle *et al.* 2012; Savelieff *et al.* 2013; Tamas *et al.* 2014).

Thorsen and colleagues identified 276 genes that were required for cadmium but not arsenite resistance in budding yeast (Thorsen *et al.* 2009). Notable GO terms that were enriched among these genes included “endosomal transport” (fourfold, 1.9e−04) “vacuole organization” (fourfold, 3.2e−04), and “vacuolar transport” (threefold, 4.1e−03). The comparable list of 70 genes in fission yeast enriched GO terms such as “post-Golgi vesicle-mediated transport” (12-fold, 2.1e−03) and “retrograde transport, endosome to Golgi” (16-fold, 7.3e−03). Although the enriched GO terms do not overlap it is worth noting that four of the budding yeast genes mapping to the GO term “endosomal transport” also are part of the “retrograde transport, endosome to Golgi” subterm. However, the strong enrichment of the retromer complex in the fission yeast list was not found in the comparable list from budding yeast. GO term analysis did not highlight metal transport from cadmium-specific gene lists from either yeast species, but the inclusion of key metal and ion transport genes in fission yeast as noted above was matched by the analysis of the corresponding gene list from *S. cerevisiae* (Thorsen *et al.* 2009). The SAGA complex, a multifunctional coactivator of transcription, was a highly enriched GO cellular component term among the genes specifically required for cadmium resistance in fission yeast. The list of genes from budding yeast that are required for cadmium resistance was not significantly enriched for SAGA components but it did contain the SAGA subunit genes *SPT7*, *HFI1*, *GCN5*, and *ADA2*, the latter two of which are homologs of genes present in the fission yeast cadmium-specific-list (Thorsen *et al.* 2009). Thus it seems likely that SAGA is important for cadmium tolerance in *S. cerevisiae* even if the screen failed to enrich SAGA subunits at a statistically significant level.

Taken as a whole these data suggest substantial conservation of toxic metal defense mechanisms between the highly divergent yeasts *S. cerevisiae* and *S. pombe*, even though GO term enrichment shows limited overlap. The data point to the utility of performing corresponding functional profiling studies with both organisms.

## Supplementary Material

Supplemental Material
