# Supplementary material for: Global Fitness Profiling Identifies Arsenic and Cadmium Tolerance Mechanisms in Fission Yeast
Source: G3 (Bethesda). 2016 Aug 22;6(10):3317–33. doi: 10.1534/g3.116.033829 (PMC5068951; doi:10.1534/g3.116.033829)
Supplement: Supplemental Material [file supp_6_10_3317__index.html]

Global Fitness Profiling Identifies Arsenic and Cadmium Tolerance Mechanisms in Fission Yeast — Supplemental Material 

# Global Fitness Profiling Identifies Arsenic and Cadmium Tolerance Mechanisms in Fission Yeast

## Supplemental Material for Guo *et al.*, 2016

**Files in this Data Supplement:**

- Figure S1 - Microculture growth assays and correlation coefficients of GI scores. (.tif, 14 MB)
- Figure S2 - Analysis of Mef2. (.tif, 8 MB)
- Figure S3 - SAGA regulates gst2 expression. (.tif, 8 MB)
- Table S1 - DNA primers used in this study. (.xlsx, 43 KB)
- Table S2 - Growth inhibition data. (.xls, 768 KB)
- Table S3 - Cadmium-sensitive mutants. (.xlsx, 83 KB)
- Table S4 - Arsenite-sensitive mutants. (.xls, 142 KB)
- Table S5 - Genes required for arsenite and cadmium resistance. (.xlsx, 84 KB)
