# Supplementary figures and images for: Global Fitness Profiling Identifies Arsenic and Cadmium Tolerance Mechanisms in Fission Yeast
Source: G3 (Bethesda). 2016 Aug 22;6(10):3317–33. doi: 10.1534/g3.116.033829 (PMC5068951; doi:10.1534/g3.116.033829)

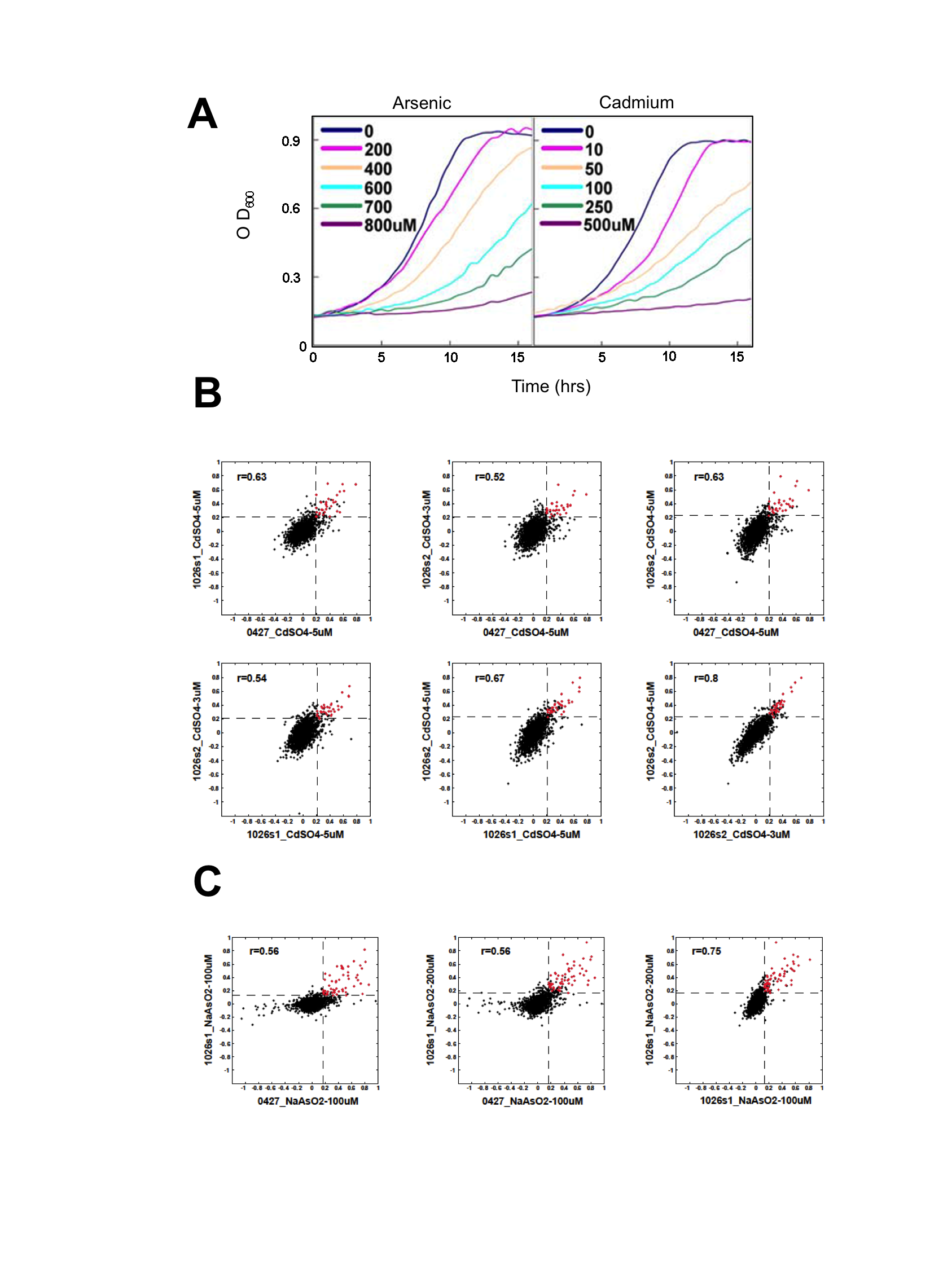

Supplement: Supplemental Material [file supp_g3.116.033829_FigureS1.tif]

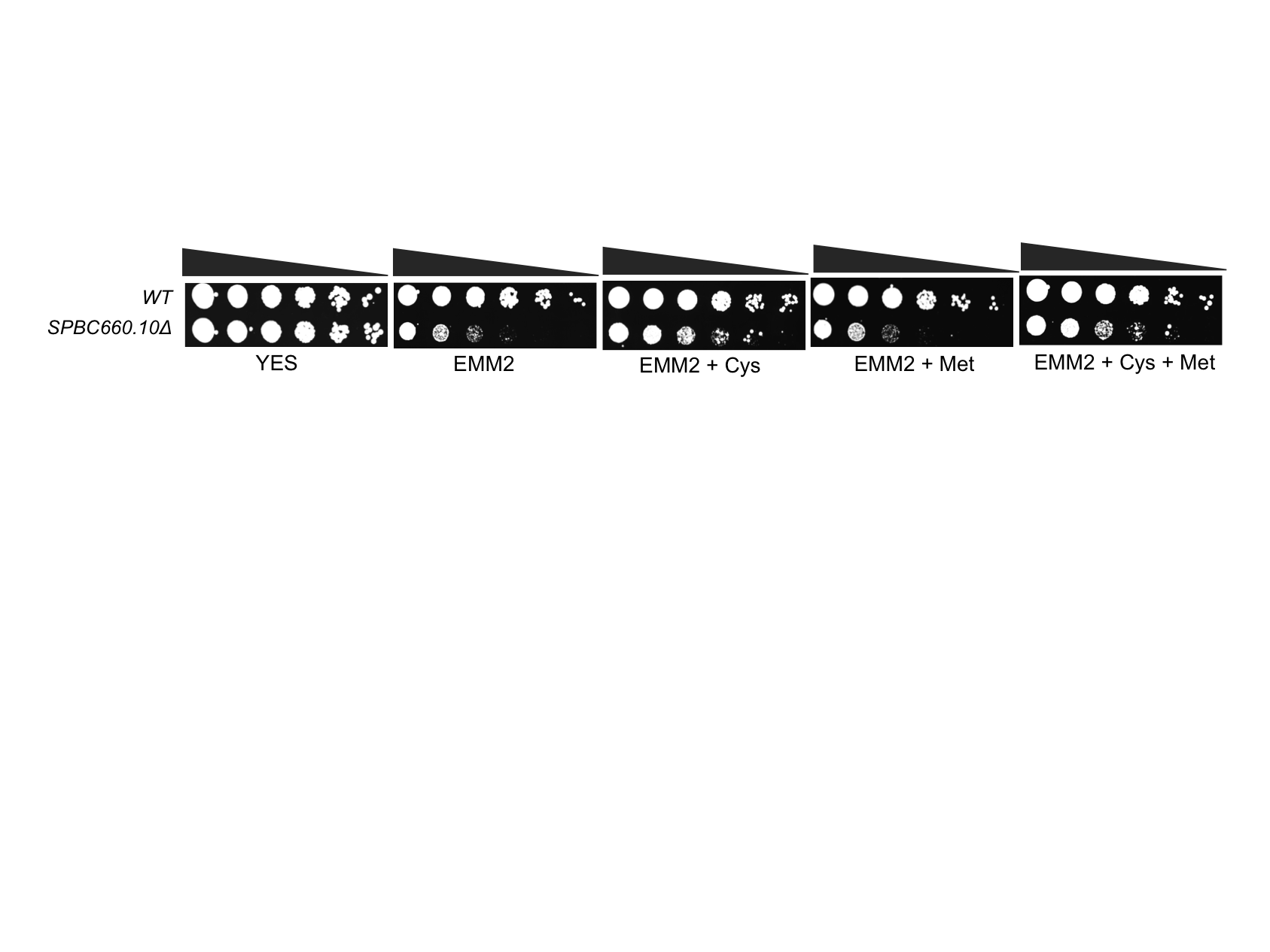

Supplement: Supplemental Material [file supp_g3.116.033829_FigureS2.tif]

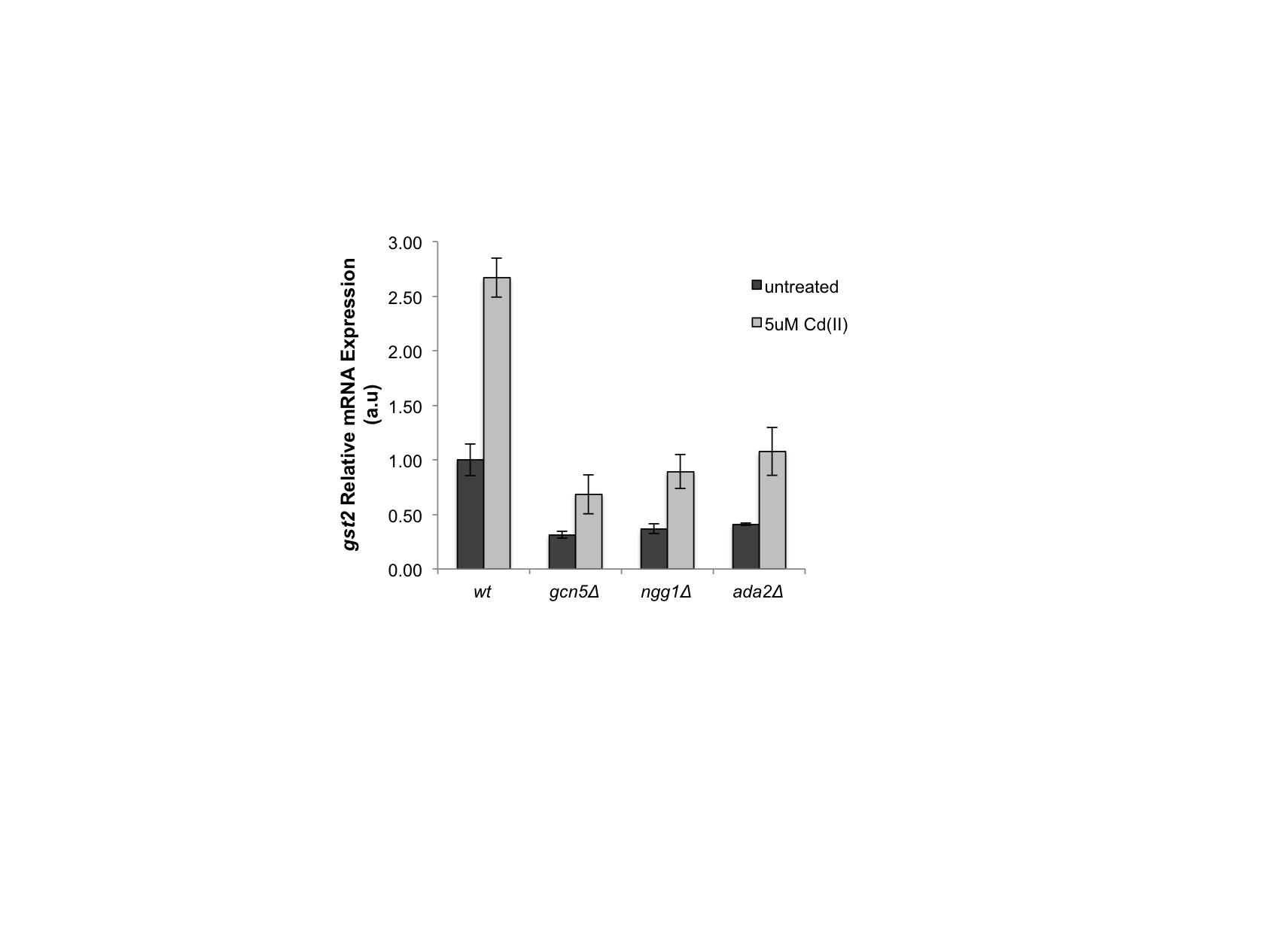

Supplement: Supplemental Material [file supp_g3.116.033829_FigureS3.tif]
